# Comparing the associations between host and tumor factors with survival outcomes with anti‐PD‐1 immunotherapy in metastatic melanoma

**DOI:** 10.1002/cam4.5070

**Published:** 2022-08-04

**Authors:** Kim Koczka, Rodrigo Rigo, Eugene Batuyong, Sara Cook, Mohammad Asad, Isabelle Vallerand, Aleksi Suo, Edwin Wang, Tina Cheng

**Affiliations:** ^1^ Department of Oncology University of Calgary Calgary Alberta Canada; ^2^ Cumming School of Medicine University of Calgary Calgary Alberta Canada; ^3^ Department of Oncology British Columbia Cancer Agency Abbotsford British Columbia Canada

**Keywords:** melanoma, prognostic, survival and immunotherapy

## Abstract

**Background:**

Anti‐programmed death‐1 (PD‐1) immunotherapy has drastically improved survival for metastatic melanoma; however, 50% of patients have progression within 6 months despite treatment. In this study, we investigated host, and tumor factors for metastatic melanoma patients treated with anti‐PD‐1 immunotherapy.

**Methods:**

Patients treated with the anti‐PD‐1 immunotherapy between 2014 and 2017 were identified in Alberta, Canada. All patients had Stage IV melanoma. Patient characteristics, investigations, treatment, and clinical outcomes were obtained from electronic medical records.

**Results:**

We identified 174 patients treated with anti‐PD‐1 immunotherapy. At 37.1 months median follow‐up time 135 (77.6%) individuals had died and 150 (86.2%) had progressed. An elevated lactate dehydrogenase (LDH) had a response rate of 21.0% versus 41.0% for those with a normal LDH (*p* = 0.017). Host factors associated with worse median progression‐free survival (mPFS) and median overall survival (mOS) included liver metastases, >3 sites of disease, elevated LDH, thrombocytosis, neutrophilia, anemia, lymphocytopenia, and an elevated neutrophil/lymphocyte ratio. Primary ulcerated tumors had a worse mOS of 11.8 versus 19.3 months (*p* = 0.042). We identified four prognostic subgroups in advanced melanoma patients treated with anti‐PD‐1 therapy. (1) Normal LDH with <3 visceral sites, (2) normal LDH with ≥3 visceral sites, (3) LDH 1‐2x upper limit of normal (ULN), (4) LDH ≥2x ULN. The mPFS each group was 14.0, 6.5, 3.3, and 1.9 months, while the mOS for each group was 33.3, 15.7, 7.9, and 3.4 months.

**Conclusion:**

Our study reports that host factors measuring the general immune function, markers of systemic inflammation, and tumor burden and location are the most prognostic for survival.

## Graphical abstract

Kaplan–Meier curve of performance‐free survival of cutaneous and melanoma of unknown primary patients stratified by prognostic criteria. LDH, lactate dehydrogenase; mPFS, median performance survival; ULN, upper limit of normal.

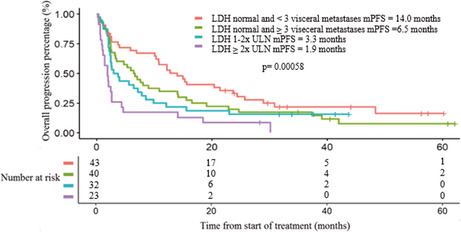

## 1 BACKGROUND

Two systemic therapeutic approaches have improved survival in metastatic melanoma over the past decade. Treatment with v‐Raf murine sarcoma viral oncogene homolog B (BRAF) inhibitors and mitogen‐activated protein kinase (MEK) inhibitors can induce rapid tumor control with improved overall survival (OS) in BRAF mutant melanoma patients; however, the duration of response is often short lived.^1^, ^2^ Immune‐checkpoint blockade with anti‐cytotoxic T‐lymphocyte associated protein 4 (CTLA‐4) and anti‐programmed death‐1 (PD‐1) immunotherapy results in sustained tumor regression with higher long‐term survival rates in patients with advanced melanoma.^3^, ^4^ Anti‐PD‐1 treatment with either nivolumab or pembrolizumab induces higher response rates and substantially extends survival compared to anti‐CTLA‐4 immunotherapy with ipilimumab alone.^5^, ^6^ Combination of ipilimumab and nivolumab for metastatic melanoma further improves survival outcomes with a 5‐year survival rate of 52% compared to 44% with nivolumab monotherapy.^5^ Nonetheless, despite anti‐PD‐1 monotherapy or combined with ipilimumab, 30%–50% of metastatic patients will have progression within 6 months of treatment.^5^

Immune‐checkpoint inhibitors have no direct anti‐cancer effect but rather achieve control by overcoming cancer immune evasion by restoring a host immune response against the tumor.^3^, ^4^ To date, there are no validated biomarkers which can confidently predict the benefit of anti‐PD‐1 immunotherapy in metastatic melanoma. Biomarkers which have focused on the tumor characteristics have largely been unsuccessful. Tumors with higher levels of programmed death‐ligand 1 (PD‐L1) have been associated with improved outcomes with anti‐PD‐1 therapy, yet tumors without any PD‐L1 expression still have durable responses. In CheckMate‐067, patients lacking PD‐L1 expression still had superior survival outcomes with nivolumab compared to ipilimumab, indicating a more complex immune response than just PD‐1 interaction with PD‐L1. A higher tumor mutational burden (TMB) has also been retrospectively shown to have higher response rates and superior OS, but individuals with low TMB still have responses to anti‐PD‐1 therapy. We hypothesize that effective anti‐cancer responses from immunotherapy are dependent on both host and tumor factors. In this study, we performed a comprehensive analysis of survival outcomes associated with clinical and histopathological parameters for patients treated with anti‐PD‐1 monotherapy.

## 2 METHODS

### 2.1 Patients

We retrospectively identified all adult individuals with metastatic melanoma treated with either nivolumab or pembrolizumab in the Province of Alberta, Canada between June 2014 and May 2017. Patients were identified using a provincial pharmacy database. The Health Research Ethics Board of Alberta Cancer Committee approved this study.

### 2.2 Treatment and assessment

Pembrolizumab was dosed at 2 mg/kg intravenously every 3 weeks, and nivolumab at 3 mg/kg intravenously every 2 weeks. Treatment continued until progression, intolerable toxicity, patient decision, or clinical decision to stop treatment. Radiological response was typically assessed every 8–12 weeks at the discretion of the treating medical oncologist, with either computed tomography (CT), positron emission tomography (PET)‐CT, or magnetic resonance imaging (MRI). Objective responses were determined for each patient as complete response (CR), partial response (PR), stable disease (SD), or progressive disease (PD) using the Response Evaluation Criteria in Solid Tumors (RECIST) 1.1 criteria.^7^ Disease control rate was defined as percentage of patients with CR, PR, or SD maintained for >6 weeks. Median follow‐up was calculated from censored patients from initiation of anti‐PD‐1 immunotherapy to last clinic assessment.

### 2.3 Analysis

We extracted patient characteristics, investigations, imaging, and clinical outcomes from electronic medical records. We obtained all routinely available clinical and pathological parameters at baseline prior to treatment initiation. Only patients with measurable disease and histologically confirmed melanoma were included in our analysis. The American Joint Committee on Cancer (AJCC) 8th edition was used for staging.^8^ Melanoma types were classified using The World Health Organization (WHO) Classification of Skin Tumors 4th edition.^9^ High chronic sun damaged (CSD) melanomas were evaluated based on histological findings of ultraviolet (UV) skin damage: Desmoplastic, lentigo melanoma, or solar elastosis, primary melanoma arising from the head and neck or distal extremities with an age above 55, and the absence of BRAF mutation. Low‐CSD melanoma was defined as the lack of solar elastosis on pathology, primary site at the truncal and proximal extremities, or presence of BRAF mutation. BRAF mutant melanoma included V600E and V600K mutations. The cut‐off values for lower limit of normal or upper limit of normal (ULN) were obtained from each individual laboratory.

Our primary objective was to identify baseline host and tumor factors that were associated with OS from the initiation of anti‐PD‐1 therapy. Secondary objectives were to identify host and tumor factors associated with ORR, and progression‐free survival (PFS). Given that molecular studies have demonstrated that MUP have cutaneous genetic signatures, they were included along with the cutaneous cohort for analysis investigating baseline prognostic factors.^10^ Host factors were divided into four domains including host general health (age, ECOG, creatinine, etc.), tumor burden and metastatic involvement (lactate dehydrogenase [LDH], organ sites of metastases, number of metastases, etc.), immune function and inflammatory dysfunction (neutrophilia, thrombocytosis, etc.), and melanoma‐specific factors (BRAF mutation, BRAF/MEK targeted treatment, etc.). Tumor factors included parameters reflecting different molecular pathogenesis (location of the primary, BRAF mutational status, high‐CSD, distinct histological subtype, etc.), and histopathological morphology features (melanoma subtypes, Breslow Depth, mitosis, ulceration, etc.). Tumor factors were collected from initial surgery of the primary lesion, therefore, the MUP cohort could not be included. We performed exploratory analysis to construct prognostic or predictive subgroups for survival based on univariate and multivariate analysis.

### 2.4 Statistical analysis

Kaplan–Meier estimates were used for estimating median PFS (mPFS) and median OS (mOS) times with 95% confidence intervals (CI). mOS and mPFS were defined as the shortest time the survival probability drops to 0.5 or below on Kaplan curves. Brookmeyer–Crawley methods were used to estimate 95% CIs. We assessed the predictive and prognostic value for each individual parameter using univariate analysis. For univariate analysis, we used Cox proportional hazards model to predict hazard ratios (HRs) and 95% CIs for each factor. Multivariate models were constructed using Cox proportional hazards models by adjusting for factors that were identified as being significant (*α* = 0.05) in the univariate analysis. Patients with missing data were excluded from multivariate analysis. Patients who did not die during the observation period were censored.

In exploratory analysis of prognostic groupings, we created subgroups stratified by level of LDH elevation with the addition of number metastases based on a similar prior study by Long et al.^11^ Our prognostic model differed from Long's with number of metastases used instead of number of visceral metastases sites based on number of metastases was more statistically significant in our cohort, and the ability to separate patients in our cohort.

We then constructed multivariate Cox proportional hazards models to see if univariate variable significance was maintained when adjusted for ECOG, metastatic stage, BRAF status, and treatment line. We also tested our prognostic model subgroups in multivariate analysis to see if it remained statistically significant when adjusted for ECOG, metastatic stage, BRAF status, and line of treatment.

The proportional hazards assumption was tested using Shoenfeld residuals (no violations were detected for any of the analysis). For Kaplan–Meier analysis, we used survival (version 3.2.7) and suvminer (version 0.4.9) packages. We used R version 4.04 for our Cox proportional hazards models. All statistical analyses were based on *α* = 0.05.

## 3 RESULTS

We identified 174 patients with metastatic melanoma with measurable disease who received at least one dose of anti‐PD‐1 immunotherapy. Table 1 reports patients' demographics and baseline characteristics at the initiation of treatment. The melanoma types were: Cutaneous in 115 (66.1%) patients, MUP in 28 (16.1%), mucosal in 17 (9.8%), uveal in 12 (6.9%), and other in 2 (1.1%) patients. Of the cutaneous patients, 10 (8.7%) were acral melanoma. At the initiation of anti‐PD‐1 immunotherapy the median age was 64 years old, 101 (58%) individuals were male, and 48 patients (27.6%) had BRAF mutant melanoma. One hundred fifty‐eight (90.8%) received treatment at a tertiary center while 16 (9.2%) were treated at community centers. Metastatic stage was M1a in 33 (19.0%), M1b in 23 (13.2%), M1c in 90 (51.7%), and M1d in 28 (16.1%) patients.^8^ One hundred forty‐four (82.8%) patients were either ECOG 0 or 1.

**TABLE 1:** Patient demographics and characteristics at initiation of anti‐PD‐1 immunotherapy for entire cohort

Characteristic	All patients, *n* (%) (*n* = 174)
Median age, years (range)	64 (24–90)
<64	93 (53.4)
≥65	81 (46.6)
Sex, male	101 (58.0)
ECOG	
0	40 (23.0)
1	104 (59.8)
2	18 (10.3)
3	6 (3.4)
4	1 (0.6)
Unknown	5 (2.9)
Metastatic stage	
M_1a_	33 (19.0)
M_1b_	23 (13.2)
M_1c_	90 (51.7)
M_1d_	28 (16.1)
Melanoma type	
Cutaneous	115 (66.1)
Melanoma of unknown primary	28 (16.1)
Mucosal	17 (9.8)
Uveal	12 (6.9)
Other	2 (1.1)
BRAF mutation positive	48 (27.6)
LDH	
≤ULN	99 (56.3)
>ULN	70 (40.2)
>2X ULN	32 (18.4)
Unknown	5 (2.9)
Line of anti‐PD‐1	
1	69 (39.7)
2	37 (21.3)
3	52 (29.9)
≥4	16 (9.2)
Prior systemic therapy	105 (60.3)
Ipilimumab treatment	90 (51.7)
Chemotherapy	57 (32.8)
BRAF and/or MEK inhibitor	44 (25.3)

Abbreviations: BRAF, v‐Raf murine sarcoma viral oncogene homolog B; ECOG, Eastern Cooperative Oncology Group; LDH, lactate dehydrogenase; MEK, mitogen‐activated protein kinase; *n*, number; PD‐1, programmed death‐1; ULN, upper limit of normal.

Anti‐PD‐1 immunotherapy was first‐line in 69 (39.7%), second‐line in 37 (21.3%), and ≥ third‐line in 68 (39.1%) Prior treatment included ipilimumab in 90 (51.7%), BRAF and, or MEK targeted therapy in 44 (25.3%) patients and chemotherapy in 57 (32.8%) patients. Pembrolizumab was used in 133 (76.4%) patients and nivolumab in 37 (21.3%). Four (2.3%) received pembrolizumab followed by nivolumab for unique reasons, such as toxicity profile, but additional anti‐PD‐1 treatment after progression for salvage therapy was not practiced. The median number of cycles of anti‐PD‐1 therapy given was 7 (range: 1–60).

### 3.1 Treatment outcomes

At median follow‐up of 37.1 months, 150 (86.2%) patients had a progression event and 135 (77.6%) patients had died. Of the 174 patients in the entire cohort, 8 (4.6%) had a CR, 43 (24.7%) had a PR, 36 (20.7%) had SD, and 87 (50%) patients had disease control. The mPFS was 3.9 months and mOS was 12.4 months.

Cutaneous melanomas demonstrated the longest mPFS and mOS at 6.7 and 14.7 months (Table S1). The MUP patients had a worse PFS and OS (2.6 and 7.8 months) compared to the primary cutaneous patients, although statistically non‐significant. The mPFS for mucosal and ocular melanoma were 2.6 and 2.9 months, and the mOS was 6.9 and 8.3 months, respectively. The ORR, mPFS, and mOS for different melanoma types and histological subtypes are listed in Table S1.

### 3.2 General host health

One hundred forty‐three patients had either cutaneous melanoma or MUP and were analyzed for host factors as shown in Table 2. Patients with an ECOG of ≥1 had an mPFS of 3.7 versus 7.6 months although not statistically significant and a lower mOS of 11.7 versus 24.9 months (HR 1.74; *p* = 0.016). Age ≥65 years, male sex, BMI, and creatinine were not found to have an association with PFS or OS. There is no statistically significant difference in ORR by age, sex, and ECOG (Table S4).

**TABLE 2:** Host factors association with progression‐free survival and overall survival outcomes by univariate analysis for cutaneous and primary unknown patients

Feature	Patient (*n*)	Progression‐free survival			Overall survival		
		Median time (months)	HR (95% CI)	*p*‐value	Median time (months)	HR (95% CI)	*p*‐value
General host health							
Age							
≤64 years	77	3.9			11.9		
≥65 years	66	9.9	0.87 (0.61–1.24)	0.42	15.6	0.87 (0.59–1.27)	0.47
Sex							
Male	89	6.4			14.3		
Female	54	5.1	1.16 (0.81–1.16)	0.41	11.9	1.06 (0.58–1.59)	0.77
ECOG							
0	37	7.6			24.9		
≥1	101	3.7	1.27 (0.84–1.92)	0.25	11.7	1.74 (1.10–2.75)	0.016
BMI							
≥25	98	4.7			12.5		
<25	35	8.5	0.97 (0.64–1.47)	0.74	20.9	0.94 (0.60–1.47)	0.87
Creatinine							
0–99	113	7.1			15.8		
≥100	27	5.6	0.97 (0.62–1.54)	0.9	12.5	1.10 (0.68–1.7)	0.71
Tumor burden and metastatic involvement							
LDH							
Normal	83	10.7			20.9		
>ULN	55	2.5	1.73 (1.19–2.52)	0.0034	6.4	2.04 (1.37–3.04)	0.00036
≥2x ULN^a^	23	1.9	2.32 (1.45–3.71)	0.00031	3.4	2.60 (1.59–4.25)	<0.0001
Liver metastasis							
No	105	7.6			16.1		
Yes	38	2.4	1.66 (1.22–2.46)	0.012	6.4	1.86 (1.23–2.80)	0.0028
Brain metastasis							
No	118	4.7			12.5		
Yes	25	7.1	1.09 (0.69–1.72)	0.71	14.1	1.17 (0.73–1.89)	0.51
Bone metastasis							
No	102	6.0			14.1		
Yes	41	3.9	0.93 (0.62–1.38)	0.70	11.7	1.16 (0.77–1.77)	0.48
Total number of metastases							
<3	58	12.2			20.4		
≥3 metastases	85	3.2	1.65 (1.14–2.38)	0.0076	8.4	1.93 (1.28–2.91)	0.0013
Immune function and inflammatory dysfunction							
Hemoglobin							
>LLN	92	7.8			17.7		
<LLN	49	2.3	1.73 (1.19–2.52)	0.0035	6.1	2.02 (1.36–3.00)	0.00037
Platelets							
<ULN	131	6.4			14.7		
≥ULN	10	1.3	3.39 (1.76–6.56)	0.00013	1.3	4.81 (2.45–9.46)	<0.0001
Neutrophils							
<ULN	123	7.1			15.6		
≥ULN	19	2.1	2.43 (1.44–4.10)	0.00059	2.9	3.2 (1.88–5.45)	<0.0001
Lymphocytes							
≥1.0	100	7.6			15.6		
<1.0	42	2.5	1.33 (0.90–1.96)	0.16	6.1	1.52 (1.01–2.29)	0.044
Neutrophils to lymphocyte ratio							
>4	56	2.5			5.2		
≤4	86	8.3	0.66 (0.46–0.95)	0.025	19.4	0.50 (0.36–0.78)	0.00038
Melanoma‐specific factors							
BRAF and/or MEK treatment							
No	100	6.9			15.8		
Yes	43	2.6	1.38 (0.94–2.01)	0.099	7.2	1.44 (0.96–2.16)	0.075
Immunotherapy line of treatment							
First	53	10.8			21.0		
Second or greater	90	3.2	1.50 (1.03–2.19)	0.033	10.8	1.59 (1.06–2.40)	0.025

Abbreviations: BMI, body mass index; BRAF, v‐Raf murine sarcoma viral oncogene homolog B; CI, confidence interval; ECOG, Eastern Cooperative Oncology Group; HR, hazard ratio; LDH, lactate dehydrogenase; LLN, lower limit of normal; *n*, number; PD‐1, programmed death‐1; ULN, upper limit of normal.

^a^ Compared to LDH <2x ULN.

### 3.3 Tumor burden and metastatic involvement

Elevated LDH had a reduced PFS (2.5 vs. 10.7 months, HR1.73; *p* = 0.0034) and OS (6.4 vs. 20.9 months, HR 2.04; *p* = 0.00036) as shown in Table 2. LDH ≥2x the ULN had a PFS of 1.9 versus 7.2 months (HR2.32; *p* = 0.00032) and an OS of 3.4 versus 15.8 months (HR 2.60; *p* < 0.0001). Patient with liver metastasis had a shorted PFS of 2.4 months compared to 7.6 months (HR 1.66; *p* = 0.012) and OS of 6.4 versus 16.1 months (HR 1.86; *p* = 0.0028). Brain metastasis(es) and bone metastasis(es) were not associated with PFS or OS. Patients with ≥3 metastases of any location had a significantly worse PFS of 12.2 versus 3.2 months (HR 1.65; *p* = 0.0076), and OS of 8.4 versus 20.3 months (HR1.93; *p* = 0.0013) (Table 2). Patients with ≥2 visceral sites of metastases had a non‐significant trend toward worse PFS and a significantly shorter OS of 11.8 versus 28.1 months, (HR 1.57; *p* = 0.022), as shown in Table S2. With increasing number of sites of visceral involvement, there were further reductions in PFS and OS with stronger statistical significance (Table S2). Patients with a normal LDH had an ORR of 41.0% compared to 21.0% with an LDH ≥ the ULN (*p* = 0.017).

### 3.4 Immune function and inflammatory dysfunction

Anemia was associated with a worse PFS of 2.3 versus 7.8 months (HR 1.73; *p* = 0.0035) and OS of 6.1 months compared to 17.7 months (HR 2.02, *p* = 0.00037) (Table 2.). Thrombocytosis had a PFS of 1.3 months compared to 6.4 months (HR 3.39; *p* = 0.00013), while the OS was 1.3 versus 14.7 months (HR 4.81; *p* < 0.0001). Neutrophilia had a shorter PFS (2.1 vs. 7.1 months, HR 1.52; *p* = 0.00059) and OS (2.9 vs. 15.6 months, HR 3.2; *p* < 0.0001). Lymphocytes <1.0 were found to have a numerically shorter PFS and significantly worse OS (6.1 15.6 months, HR 01.52; *p* = 0.044). A neutrophil to lymphocyte ratio of ≥4 was associated with a worse PFS (2.5 vs. 8.3 months HR 1.52; *p* = 0.025) and OS (5.2 vs. 19.4 months HR 2.0; *p* = 0.00038). Response rates were not statistically different for anemia, neutrophilia, thrombocytosis, or neutrophil to lymphocyte ratio.

### 3.5 Melanoma‐specific factors

Patients with prior BRAF and/or MEK inhibitor targeted therapy had a trend toward worse PFS (2.6 vs. 6.9 months, HR 1.38, *p* = 0.099) and OS (7.2 vs. 15.8 months, HR 1.44; *p* = 0.075) which was not statistically significant (Table 2). Patients who were treated with first‐line anti‐PD‐1 immunotherapy had improved PFS of 10.8 months compared to 3.2 months for those treated with ≥ second‐line therapy (HR 1.50, *p* = 0.033), and superior OS 21.0 versus 10.8 months (HR 1.59, *p* = 0.025).

### 3.6 Tumor factors

Pathological parameters for 115 patients with cutaneous melanoma are shown in Table 3. BRAF mutation had a trend toward worse PFS (3.4 vs. 6.7 months) compared to BRAF wild‐type (HR 1.43, *p* = 0.054) and a non‐significant worse OS of 7.5 months compared to 14.8 months. Location of melanoma primary and CSD were not associated with either PFS of OS. The 62 patients with an ulcerated lesion had a worse mOS of 11.8 versus 19.3 months (HR 1.65, *p* = 0.042). Other histology factors including Breslow thickness, tumor‐infiltrating lymphocytes (TILs), mitosis, lesion pigmentation, or regression were not associated with PFS or OS as seen in Table 3.

**TABLE 3:** Tumor factors association with progression‐free survival and overall survival outcomes by univariate analysis for cutaneous and primary unknown patients

Feature	Patient (*n*)	Progression‐free survival			Overall survival		
		Median time (months)	HR (95% CI)	*p*‐value	Median time (months)	HR (95% CI)	*p*‐value
Biological pathways							
Primary known or unknown							
Primary known	115	6.7			14.7		
Unknown primary	28	2.6	1.44 (0.93–2.25)	0.10	7.8	1.37 (0.86–2.20)	0.18
Melanoma primary locations							
Truncal and proximal extremities	55	7.1			15.6		
Head/neck and distal extremities	56	6.5	1.36 (0.91–2.04)	0.14	13.2	1.24 (0.80–1.91)	0.34
Chronic sun damage melanoma							
Low	40	5.0			14.7		
High	73	7.2	0.81 (0.53–1.20)	0.30	15.6	0.83 (0.53–1.28)	0.39
BRAF mutation status							
Negative	94	6.7			14.8		
Positive	48	3.4	1.43 (0.99–2.08)	0.054	7.5	1.31 (0.88–1.95)	0.18
Histological subtypes							
Superficial spreading subtype							
No	119	5.3			12.4		
Yes	24	6.5	1.04 (0.65–1.67)	0.85	15.8	0.93 (0.56–1.56)	0.80
Nodular subtype							
No	116	4.7			12.0		
Yes	27	7.9	1.02 (0.65–1.56)	0.97	15.8	0.99 (0.63–1.61)	0.90
Desmoplastic subtype							
No	139	5.6			12.5		
Yes	4	NR	6.51 (0.91–46.7)	0.032	NR	4.60 (0.64–33.0)	0.095
Lentigo‐maligna subtype							
No	136	5.6			12.5		
Yes	7	22.9	2.14 (0.79–5.80)	0.12	22.9	1.49 (0.55–4.05)	0.43
Routine histology factors							
Ulceration of primary tumor							
Absent	37	7.8			19.3		
Present	62	4.1	1.3 (0.84–2.03)	0.24	11.8	1.65 (1.01–2.70)	0.042
Breslow							
T1	11	10.3			48.4		
T2‐T4	93	6.0	1.20 (0.62–2.33)	0.58	13.2	1.61 (0.73–3.55)	0.23
TIL							
Absent	26	5.8			12.0		
Present	61	5.9	0.96 (0.59–1.56)	0.86	14.7	0.83 (0.50–1.40)	0.49
Mitosis number per mm^2^							
0–1	14	6.6			14.3		
2–9	45	2.6	0.90 (0.48–1.70)		11.9	1.11 (0.55–2.27)	
>9	31	6.7	0.84 (0.43–1.64)	0.87	14.8	1.11 (0.53–2.32)	0.95
Regression							
Absent	74	6.5			13.7		
Present	19	2.6	1.23 (0.73–2.09)	0.43	6.4	1.30 (0.74–2.28)	0.36
Pigmentation							
Absent	13	6.0			14.3		
Present	72	5.6	1.25 (0.66–2.37)	0.49	12.4	1.10 (0.56–2.16)	0.79
Benign association lesion							
Absent	33	10.3			16.1		
Present	16	4.0	1.37 (0.57–3.08)	0.36	12.5	1.59 (0.68–3.74)	0.28
Cell type							
Epithelioid	29	10.3			24.2		
Spindle or Mixed	19	6.9	1.35 (0.71–2.56)	0.1	16.1	1.68 (0.83–3.38)	0.15

Abbreviations: BRAF, v‐Raf murine sarcoma viral oncogene homolog B; CI, confidence interval; HR, hazard ratio; *n*, number; TIL, tumor‐infiltrating lymphocytes.

Different histology subtypes are reported in Table 3. Desmoplastic patients had a trend toward improved mOS, which did not meet statistical significance (not reached vs. 12.5 months; HR 4.60, *p* = 0.095). Superficial spreading, nodular, and lentigo melanoma had no difference in mPFS or OS. Response rate in desmoplastic melanoma and other histological subtypes are summarized in Table S1.

### 3.7 Multivariate analysis

Host factors and tumor factors that had a statistically significant OS were included in a multivariate model and are reported in Table 4. Statistical significance for OS was maintained for ≥3 sites of metastases (*p* = 0.046), LDH 1‐2X elevation (*p* = 0.05), LDH ≥2X ULN elevation (*p* = 0.001), anemia (*p* = 0.002), thrombocytosis (*p* ≤ 0.001), neutrophilia (*p* ≤ 0.001), lymphocytes below <1.0 (*p* = 0.043), and presence of liver metastases (*p* = 0.007). Statistically significant association for OS was not maintained with multivariate analysis for ECOG ≥1 (*p* = 0.075) and presence of ulceration in the primary (*p* = 0.076).

**TABLE 4:** Multivariate analysis of host and tumor factors association with progression‐free survival and overall survival for cutaneous and primary unknown patients

Feature	Patients (*n*)	Progression‐free survival			Overall survival		
		HR	95% CI	*p*‐value	HR	95% CI	*p*‐value
ECOG							
0	37	–	–	–	–	–	–
≥1	101	1.24	0.81–1.90	0.30	1.53	0.96–2.45	0.075
LDH							
Normal	83	–	–	–	–	–	–
1–2X ULN	55	1.58	0.97–2.58	0.067	1.67	1.00–2.79	0.050
≥2x ULN	23	2.41	1.45–4.45	0.001	2.71	1.49–4.94	0.001
Liver metastasis							
No	105	–	–	–	–	–	–
Yes	38	1.87	1.15–3.02	0.011	1.98	1.20–3.27	0.007
Total metastases							
< 3	58	–	–	–	–	–	–
≥3 metastases	85	1.54	0.99–2.41	0.056	1.61	1.01–2.58	0.046
Hemoglobin							
>LLN	92	–	–	–	–	–	–
<LLN	49	1.72	1.15–2.56	0.008	1.96	1.28–3.01	0.002
Platelets							
<ULN	131	–	–	–	–	–	–
≥ULN	10	3.26	1.57–6.77	0.002	3.99	1.91–8.37	<0.001
Neutrophils							
<ULN	123	–	–	–	–	–	–
≥ULN	19	2.83	1.62–4.94	<0.001	3.67	2.10–6.43	<0.0.001
Lymphocytes							
≥1.0	100	–	–	–	–	–	–
<1.0	42	0.73	0.48–1.11	0.14	0.64	0.42–0.99	0.043
Neutrophils to lymphocyte ratio							
>4	56	–	–	–	–	–	–
≤4	86	1.56	1.06–2.31	0.024	2.01	1.33–3.04	<0.001
Ulceration of primary tumor							
Absent	37	–	–	–	–	–	–
Present	62	1.28	0.77–2.12	0.3	1.64	0.95–2.83	0.076

Abbreviations: BRAF, v‐Raf murine sarcoma viral oncogene homolog B; CI, confidence interval; ECOG, Eastern Cooperative Oncology Group; HR, hazard ratio; LDH, lactate dehydrogenase; LLN, lower limit of normal; *n*, number; TIL, tumor infiltrating lymphocytes; ULN, upper limit of normal.

### 3.8 Prognostic grouping

**FIGURE 1:**
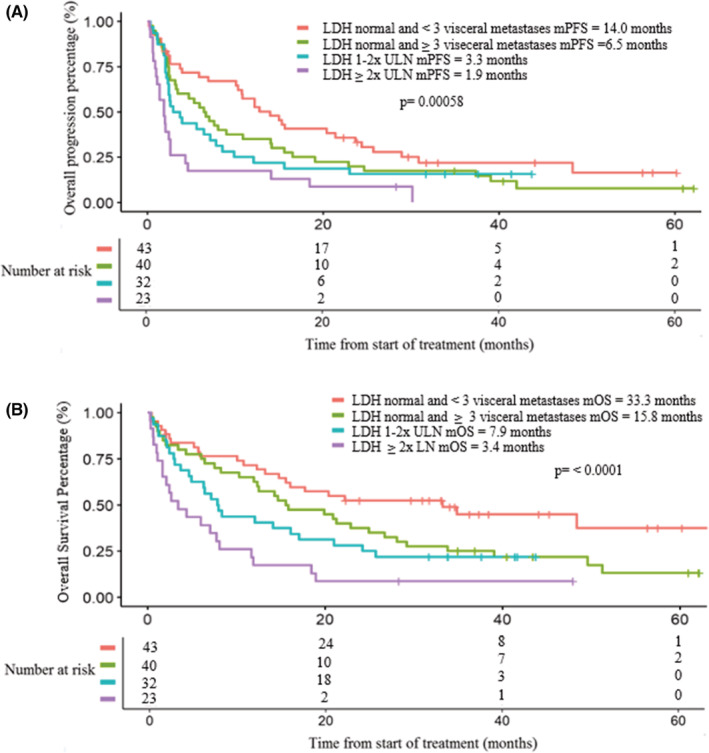
Kaplan–Meier curve of (A) performance‐free survival and (B) overall survival of cutaneous and melanoma of unknown primary patients stratified by prognostic criteria. LDH, lactate dehydrogenase; mPFS, median performance survival; ULN, upper limit of normal.

In exploratory analysis, we used LDH and number of sites of disease to separate patients into four subgroups: (1) normal LDH and <3 sites of metastases; (2) normal LDH and ≥3 sites of metastases; (3) LDH 1‐2x ULN, and (4) LDH ≥2x ULN. mPFS were 14.0, 6.5, 3.3, and 1.9 months, and mOS were 33.3, 15.7, 7.9, and 3.4 months for the four subgroups, respectively (Figure 1A,B).

The four prognostic groups were adjusted for potentially confounding variables using multivariate analysis. Multivariate analysis showed a statistically significant inferior PFS for subgroups 3 (HR 1.92; *p* = 0.028) and 4 (HR 2.96; *p* < 0.001) relative prognostic Group 1, which served at the referent group (Table 5). Similarly, OS were statistically significant for Subgroup 3 (HR 2.17; *p* = 0.016) and Subgroup 4 (HR 3.20; *p* < 0.001) in comparison to Subgroup 1. Subgroups 1 and 2 both had normal LDH and the survival differences were not statistically significant. Response rates by subgroups are summarized in Table S3.

**TABLE 5:** Multivariate analysis of melanoma prognostic subgroups for cutaneous and primary unknown patients

Prognostic group	Progression‐free survival			Overall survival		
	HR	95% CI	*p*‐value	HR	95% CI	*p*‐value
Normal LDH and <3 visceral metastases	1.00 (reference)	–	–	1.00 (reference)	–	–
Normal LDH and ≥3 visceral metastases	1.41	0.84–2.37	0.2	1.58	0.89–2.81	0.12
LDH 1‐2x ULN	1.92	1.07–3.42	0.028	2.17	1.15–4.07	0.016
LDH ≥2x ULN	2.96	1.57–5.58	<0.001	3.20	1.60–6.35	<0.001

Abbreviations: CI, confidence interval; HR, hazard ratio; LDH, lactate dehydrogenase, ULN, upper limit of normal.

## 4 DISCUSSION

To our knowledge, this is the first study that conducted a comprehensive analysis of tumor factors or provide an in‐depth analysis of both host and tumor factors associated with survival outcomes for anti‐PD‐1 immunotherapy. Our study reports that host factors measuring general immune function, tumor burden and location, and markers of systemic inflammation are the most prognostic for survival. These include lymphopenia, neutrophilia, elevated neutrophil to leukocyte ratio, elevated LDH, number of metastases, liver metastases, thrombocytosis, and anemia. Among these, only LDH was associated with tumor response, and this is the first report we are aware of indicating that LDH is predictive of response. Using LDH, and the number of metastases, we constructed a prognostic model that is highly discriminating for survival.

We are surprised to find that tumor factors were mostly not associated with survival and that only tumor ulceration was significantly associated with OS in univariate analysis. BRAF mutation, high‐CSD melanoma, tumor regression, mitosis, TILs, and MUP were not associated with either PFS or OS. Melanoma is immunogenic cancer due to UV‐induced high TMB, resulting in neoantigens that can be recognized as foreign by the immune system.^12^, ^13^ High‐CSD melanomas typically develop on the head and neck or distal extremities in older individuals, and are usually not associated with BRAF mutations.^9^ High‐CSD melanomas have a threefold higher TMB than low‐CSD melanomas,^12^ but we found that neither high‐CSD nor BRAF mutant melanoma were associated with survival. This finding is consistent with prior reports that melanoma with a “low” TMB also respond to immunotherapy and there is only a modest difference in clinical benefit between low‐TMB and high‐TMB treated with anti‐PD1 immunotherapy for metastatic melanoma.^14^ In other solid tumors, the KEYNOTE‐158 trial showed that patients with various metastatic solid tumors with high TMB (≥10 mutations per megabase) had an ORR of 29% while patients with low TMB (<10 mutations per megabase) had an ORR of 6%.^15^ The responses in KEYNOTE‐158 were durable; however, this trial did not include melanoma patients and raises the question of what is the optimal cut off point for TMB can be used for each solid tumor in the study. At this time for metastatic melanoma patients, there are currently no biomarkers derived from the mechanism of anti‐PD‐1 immunotherapy reliably able to predict clinical benefit, including TMB, tumor‐specific neoantigens, PD‐L1 expression and infiltrating immune cells.^16^, ^17^

The “cancer‐immune set point” defines the equilibrium between factors promoting or suppressing cancer eradication.^17^ As anti‐PD‐1 immunotherapy can only achieve anti‐cancer effect through altering the host immune system, it is likely that the host immune system is responsible for surpassing the threshold to mount a sufficient immune response against cancer. The importance of host factors rather than tumor factors on treatment outcomes is consistent with the mechanism of action of immunotherapy. This is supported by the improved survival outcomes seen in advanced melanoma patients who have immune‐related adverse events (IRAE) from immunotherapy.^18^ IRAE are one of the strongest predictors to response and survival. In a retrospective prognostic study looking at melanoma or non‐small cell lung cancer NSCLC, the response rate for patients with IRAE were 53.9% versus 12.9% or patients without IRAE (*p* < 0.001), and median survival was significantly prolonged (5.3 vs. 28.2 months, *p* < 0.001) reinforcing the fact that the host immune system is integral for cancer control.^19^

For host factors associated with survival, only elevated LDH was predictive of response. Elevated LDH is an independent prognosticator of poor survival in metastatic melanoma,^8^ and remains prognostic for advanced melanoma patients treated with ipilimumab, anti‐PD‐1immunotherapy, and dabrafenib with trametinib.^11^, ^20^, ^21^ In our study, patients with a normal LDH had an ORR of 41.0% versus 20.0% for LDH > ULN (*p* = 0.017) as shown in Table S4. Similarly, in KEYNOTE‐001, response rates for advanced melanoma patients treated with single‐agent pembrolizumab were 42% with a normal LDH and 22.3% with an elevated LDH.^22^ These findings suggest that an elevated LDH predicts a suppressed and impaired response to anti‐PD‐1 immunotherapy.

In melanoma, individual biomarkers represent an individual component of a dynamic cancer‐immune interaction, and alone are unable to reliably predict responses. The cancer immunogram described by Blank et al is a framework describing the essential component for an effective immune response against the malignancy.^23^ The proposed “cancer immunogram” consists of seven parameter classes: Tumor foreignness, general immune status, immune cell infiltration, absence of checkpoints, absence of soluble inhibitors, absence of inhibitory tumor metabolism, and tumor sensitivity to immune effectors. Elevated LDH is a marker of altered glycolysis resulting in higher lactic acid levels and lower local pH that impairs T‐cell function, cytokine production, and other immune effector functions, providing an explanation as a biomarker of inhibitory tumor metabolism.^24^ Lymphopenia, neutrophilia, and elevated neutrophil to leukocyte ratio are markers of impaired general immune status and resulted in worse survival. Systemic inflammation measured by neutrophilia, thrombocytosis, and an elevated neutrophil to leukocyte ratio represents a disordered immune function. Our study supports previous studies demonstrating reduced response rate and survival in individuals with liver metastases but not other organ sites.^25^ In Keynote‐001, only liver metastases were associated with lower rate of CR.^26^ Several mechanisms explaining decreased immunotherapy effectiveness in the liver have been proposed, including the liver trapping and destroying activated CD8+ T‐cells and expression of anti‐inflammatory cytokines resulting in inhibitory tumor metabolism.^27^, ^28^ While a coordinated inflammatory response of growth factors, cytokines, neutrophils, macrophages, and fibroblasts are needed to eradicate cancer cells, sustained cancer‐induced inflammation results in immune dysfunction and is well described to have poorer survival outcomes.^29^, ^30^ Our study suggests that markers of cancer‐associated systemic inflammation including neutrophilia, thrombocytosis, anemia and are strong predictors of worse survival when treated with anti‐PD1 immunotherapy.

We applied Long's BRAF melanoma prognostic approach to our cohort based on our findings that LDH and number of metastases were both highly associated with survival.^11^ Our model differed with number of metastases used instead of number of visceral metastases sites. The model remains highly discriminatory for survival; however, we noted significant disparity in response rates to treatment between our study and Long's meta‐analysis. Long showed that responses to dual BRAF and MEK inhibitors in patients with an elevated LDH ≥1 to <2x and LDH ≥2x ULN were still maintained (58% and 50% respectively) when compared to 78% for normal LDH and <3 visceral metastases, and 68% for normal LDH and ≥3 visceral metastases.^11^ Our study showed that response rates for patients with an elevated LDH ≥1 to <2x and LDH ≥2x ULN were markedly lower at 21.9% and 17.4%, compared to response rates of 44.2% and 37.5% for patients with normal LDH and either <3 metastases or ≥3 metastases (Table 3). The difference in response rates supports the differing mechanism of action of combined BRAF and MEK inhibitors directly inhibiting cancer cells compared to anti‐PD‐1 immunotherapy altering the host immune system. This prognostic model can be readily applied to patients in a clinical setting but needs to be validated in an independent cohort.

Our results must be interpreted with caution given the retrospective nature and small sample size. Our limited sample size, especially for histology may be underpowered to detect survival outcomes for primary tumor factors. We also have inferior survival data to prospective studies with single anti‐PD1 immunotherapy. An important reason for this inferior survival is that this population was heavily pretreated, with advanced metastatic disease. Over 60% of patients received anti‐PD1 immunotherapy as ≥ second‐line treatment, and only around 51% of the analyzed patient population had a normal LDH.

## 5 FUTURE DIRECTION

Further improvement in predicting response to immunotherapy will require incorporation of essential components of a dynamic tumor immune system based on the concept of the “cancer immunogram”. Our study suggests that cancer‐associated inflammation may present an additional component to the cancer immunogram. Strategic targeting of cancer‐associated systemic inflammation may be as important as enhancing adaptive immunity. Research targeting immune‐suppressive tumor metabolism remains ongoing.^31^, ^32^ A recent phase II trial combining ipilimumab and nivolumab with the interleukin‐6 antibody (a pro‐inflammatory cytokine) tocilizumab has shown early promise.^33^

## 6 CONCLUSION

Our study reports host factors measuring general immune function, tumor burden and location and markers of systemic inflammation are the most prognostic for survival. Among these, only LDH was associated with response, suggesting that an elevated LDH predicts a suppressed and impaired response to anti‐PD‐1 immunotherapy. Markers of cancer‐associated systemic inflammation including neutrophilia, thrombocytosis, and anemia are strong predictors of worse survival when treated with anti‐PD1 immunotherapy. Tumor histology played a much smaller role with only tumor ulceration associated with worse survival, and some specific melanoma subtypes are likely to benefit more than others. Our findings provide evidence that the host immune system is responsible for surpassing a threshold to mount a sufficient immune response against cancer. Using LDH and number of sites of metastases, we can objectively prognosticate patient outcomes on anti‐PD1 immunotherapy. This model if validated, can help clinical decision making in treating advanced melanoma.

## AUTHOR CONTRIBUTIONS

Conceptualization, Kim Koczka, Rodrigo Rigo, Aleksi Suo, and Tina Cheng; software and formal analysis, Mohammad Asad and Isabelle Vallerand; resources, Edwin Wang, Rodrigo Rigo, Eugene Batuyong, and Tina Cheng; writing—original draft preparation, Kim Koczka; writing—review and editing, Kim Koczka, Sara Cook, Tina Cheng, and Aleksi Suo; supervision, Tina Cheng, Edwin Wang. All authors have read and agreed to the published version of the manuscript.

## FUNDING INFORMATION

There was no financial support in writing this manuscript.

## CONFLICT OF INTEREST

There are no conflicts of interest to disclose.

## Supporting information


Table S1
Table S2
Table S3
Table S4

## Data Availability

NA.
