# Supplementary material for: Comparing the associations between host and tumor factors with survival outcomes with anti‐PD‐1 immunotherapy in metastatic melanoma
Source: Cancer Med. 2022 Aug 4;12(3):2427–39. doi: 10.1002/cam4.5070 (PMC9939151; doi:10.1002/cam4.5070)
Supplement: Supplementary file 1 — Table S1 Table S2 Table S3 Table S4 [file CAM4-12-2427-s001.docx]

**Table 1S. Melanoma type, subtype and response rates for entire cohort**

| Characteristics | N (%) | ORR, N (%) | CR, N (%) | mPFS (months) | mOS (months) |
| --- | --- | --- | --- | --- | --- |
| ALL  Primary Type  Cutaneous  Mucosal  Ocular  Melanoma unknown primary  Other | 174 (100)  115 (66.1)  17 (9.8)  12 (6.9)  28 (16.1)  2 (1.1) | 51 (29.3)  37 (32.2)  3 (17.6)  0 (0)  10 (35.7)  1 (50) | 8 (4.6)  6 (5.2)  1 (5.9)  0 (0)  1 (3.6)  0 (0) | 3.9  6.7  2.6  2.9  2.6  2.3 | 12.4  14.7  6.9  8.3  7.8  8.0 |
| Histological subtype  Superficial spreading  Acral  Lentigo Maligna  Nodular  Desmoplastic  Malignant Melanoma NOS  Other  Unknown | 115  24 (20.9)  10 (8.7)  7 (6.1)  26 (22.6)  4 (3.5)  33 (28.7)  4 (3.5)  7 (6.1) | 37 (32.2)  6 (25)  2 (20)  3 (42.9)  10 (38.5)  3 (75)  11 (33.3)  0 (0)  2 (28.6) | 6 (5.2)  1 (4.2)  0 (0)  0 (0)  1 (3.8)  0 (0)  2 (6.1)  0 (0)  2 (28.6) | 6.7  6.5  2.3  22.9  7.8  NR  3.7  2.3  3.5 | 14.7  15.8  9.5  22.9  15.8  NR  10.3  8.0  7.0 |

CR, complete response; mPFS, median progression free survival; mOS, median overall survival; n, number; NOS, not other wise specified.

**Table 2S: Tumour Burden Univariate Association With Progression**

**Free Survival and Overall Survival for Cutaneous and Primary Unknown Patients**

|  |  | **Progression Free Survival** | | | | **Overall Survival** | | |
| --- | --- | --- | --- | --- | --- | --- | --- | --- |
|  | **Patient (n)** | **Median time (months)** | **HR (95% CI)** | **P-value** | **Median time (months)** | | **HR (95% CI)** | **p-value** |
| <2 visceral sites  ≥2 visceral sites | 47  96 | 10.8  3.8 | 1.31 (0.89-1.92) | 0.17 | 28.1  11.8 | | 1.67 (1.08-2.58) | 0.019 |
| < 3 visceral sites  ≥ 3 visceral sites | 94  49 | 7.2  2.6 | 1.34 (0.92-1.94) | 0.12 | 15.6  6.4 | | 1.57 (1.06-2.32) | 0.022 |
| < 4 visceral sites  ≥ 4 visceral sites | 120  23 | 6.4  2.0 | 1.26 (0.78-2.04) | 0.35 | 14.7  3.5 | | 1.66 (1.02-2.70) | 0.04 |
| <5 visceral sites  ≥5 visceral sites | 134  9 | 6.4  1.1 | 3.44 (1.72-6.91) | 0.00024 | 14.7  1.7 | | 3.92 (1.95-7.87) | <0.001 |
| < 6 visceral sites  ≥ 6 visceral sites | 138  5 | 5.9  0.99 | 4.05 (1.62-10.1) | 0.0013 | 14.3  1.7 | | 5.91 (2.32-15.0) | <0.0001 |

CI, confidence interval; HR, hazard ratio; n, number.

**Table 3S Melanoma Prognostic Subgroups Response Rates and**

**Survival Outcomes for Cutaneous and Primary Unknown Patients**

| **Prognostic Group** | **Patients (n)** | **Progression events (n)** | **Deaths (n)** | **CR**  **(%)** | **ORR (%)** | **DCR**  **(%)** | **mPFS**  **(months)** | **mOS (months)** |
| --- | --- | --- | --- | --- | --- | --- | --- | --- |
| All | 138 | 121 | 106 | 7 (5.1) | 45 (32.6) | 75 (54.3) | - | - |
| Normal LDH and <3 visceral metastases | 43 | 33 | 26 | 4 (14.4) | 19 (44.2) | 31 (72.1) | 14.0 | 33.3 |
| Normal LDH and ≥ 3 visceral metastases | 40 | 36 | 34 | 2 (5.9) | 15 (37.5) | 24 (60.0) | 6.5 | 15.7 |
| LDH 1-2x ULN | 32 | 27 | 25 | 1 (3.7) | 7 (21.9) | 15 (46.9) | 3.3 | 7.9 |
| LDH ≥2x ULN | 23 | 22 | 21 | 0 (0) | 4 (17.4) | 5 (8.7) | 1.9 | 3.4 |

CR, complete response; DCR, disease control rate; mOS, LDH, lactate dehydrogenase; median overall survival; mPFS, median progression free survival; n, number; ORR, overall response rate; ULN, upper limit of normal.

**Table 4S.Host Factors and Tumour Factors Response Rates Cutaneous and Primary Unknown Patients**

| **Feature** | **Patients (n)** | **Progression events (n)** | **Deaths (n)** | **CR** | **ORR** | **DCR** | **p-value*** |
| --- | --- | --- | --- | --- | --- | --- | --- |
| Age  < 65  ≥ 65 | 77  60 | 65  51 | 59  44 | 5  2 | 27.2  39.3 | 42  33 | 0.17 |
| Sex  Male  Female | 89  54 | 78  43 | 69  39 | 4  3 | 31.4  35.2 | 48  29 | 0.78 |
| BRAF Status  Wild Type  Mutant | 94  48 | 77  43 | 68  39 | 4  3 | 37.2  25.0 | 54  22 | 0.072 |
| LDH  Normal  ≥1X ULN  ≥2X ULN* | 83  55  23 | 69  49  22 | 56  47  21 | 7.2  1.8  0 | 41.0  20.0  17.4 | 66.3  36.4  21.7 | 0.017  0.18 |
| Hemoglobin  ≥ LLN  < LLN | 92  49 | 77  44 | 64  42 | 4.3  6.1 | 38.0  22.4 | 63.0  38.6 | 0.09 |
| Neutrophils  < ULN  > ULN | 123  19 | 105  17 | 90  17 | 5.7  0 | 35.8  10.5 | 57.7  26.3 | 0.054 |
| Platelets  < ULN  > ULN | 131  10 | 111  10 | 96  10 | 5.3  0 | 34.3  10.0 | 55.7  33.3 | 0.22 |
| Melanoma Location  Truncal  Distal | 55  56 | 44  51 | 39  44 | 10.9  0 | 41.8  23.2 | 63.6  51.8 | 0.059 |
| Liver  No  Yes | 105  38 | 86  35 | 74  34 | 5.7  2.6 | 36.2  23.7 | 61.0  34.2 | 0.23 |
| ≥ 3 sites of disease  Absent  Present | 58  85 | 46  77 | 35  73 | 8.6  2.4 | 41.4  27.1 | 69.0  41.2 | 0.11 |
| Neutrophils to lymphocyte ratio  >4  ≤4 | 56  86 | 48  74 | 46  61 | 1.8  7.0 | 23.2  38.4 | 39.2  64.0 | 0.089 |
| Ulceration  Absent  Present | 37  62 | 31  54 | 24  51 | 8.1  1.6 | 29.7  30.1 | 83.8  38.7 | 1.0 |
| ECOG  0  ≥1 | 37  101 | 31  88 | 25  79 | 8.1  4.0 | 40.5  30.7 | 73.0  47.5 | 0.38 |

BRAF, v-Raf murine sarcoma viral oncogene homolog B; CR, complete response; DCR, disease control rate, LDH, lactate

dehydrogenase; LLN, lower limit of normal;N, number; ORR, overall response rate; ULN, upper limit of normal.

*p value calculated for ORR.
